# Seasonal Factors Are Associated with Activities of Enzymes Involved in High-Density Lipoprotein Metabolism among Pregnant Females in Ghana

**DOI:** 10.1016/j.cdnut.2023.102041

**Published:** 2023-11-23

**Authors:** Brian V Hong, Jack Jingyuan Zheng, Eduardo Z Romo, Joanne K Agus, Xinyu Tang, Charles D Arnold, Seth Adu-Afarwuah, Anna Lartey, Harriet Okronipa, Kathryn G Dewey, Angela M Zivkovic

**Affiliations:** 1 Department of Nutrition, University of California, Davis, Davis, CA, United States; 2 Department of Nutrition and Food Science, University of Ghana, Legon, Ghana; 3 Department of Nutritional Sciences, Oklahoma State University, Stillwater, OK, United States

**Keywords:** cholesterol efflux capacity, CETP activity, Ghana, HDL, LCAT activity, PLTP activity, pregnancy, SQ-LNS

## Abstract

**Background:**

Small-quantity lipid-based nutrient supplements (SQ-LNS) during pregnancy and postnatally were previously shown to improve high-density lipoprotein (HDL) cholesterol efflux capacity (CEC) and length in the children of supplemented mothers at 18 mo of age in the International Lipid-Based Nutrient Supplements (iLiNS) DYAD trial in Ghana. However, the effects of SQ-LNS on maternal HDL functionality during pregnancy are unknown.

**Objective:**

The goal of this cross-sectional, secondary outcome analysis was to compare HDL function in mothers supplemented with SQ-LNS vs. iron and folic acid (IFA) during gestation.

**Methods:**

HDL CEC and the activities of 3 HDL-associated enzymes were analyzed in archived plasma samples (*N* = 197) from a subsample of females at 36 weeks of gestation enrolled in the iLiNS-DYAD trial in Ghana. Correlations between HDL function and birth outcomes, inflammatory markers C-reactive protein (CRP) and alpha-1-acid glycoprotein (AGP), and the effects of season were explored to determine the influence of these factors on HDL function in this cohort of pregnant females.

**Results:**

There were no statistically significant differences in HDL CEC, plasma lecithin-cholesterol acyltransferase (LCAT) activity, cholesteryl ester transfer protein (CETP) activity, or phospholipid transfer protein (PLTP) activity between mothers supplemented with SQ-LNS compared with IFA control, and no statistically significant relationships between maternal HDL function and childbirth outcomes. LCAT activity was negatively correlated with plasma AGP (R = -0.19, *P* = 0.007) and CRP (R = -0.28, *P* < 0.001), CETP and LCAT activity were higher during the dry season compared to the wet season, and PLTP activity was higher in the wet season compared to the dry season.

**Conclusions:**

Mothers in Ghana supplemented with SQ-LNS compared with IFA during gestation did not have measurable differences in HDL functionality, and maternal HDL function was not associated with childbirth outcomes. However, seasonal factors and markers of inflammation were associated with HDL function, indicating that these factors had a stronger influence on HDL functionality than SQ-LNS supplementation during pregnancy.

**Clinical Trial Registry number:**

The study was registered as NCT00970866. https://clinicaltrials.gov/study/NCT00970866

## Introduction

Inadequate nutrient intake during pregnancy, often due to limited access to safe and nutritious foods, is one of many factors that put females in low- and middle-income countries at risk for adverse pregnancy outcomes and their children at risk for long-term negative effects on health and development [[1], [2], [3]].

Small-quantity lipid-based nutrient supplements (SQ-LNS) were developed to help prevent maternal and child malnutrition in populations whose typical diets fall far short of meeting nutrient requirements [4,5]. SQ-LNS provide multiple micronutrients and essential fatty acids, including linoleic and α-linolenic acid (ALA), to enrich the diet and complement home-prepared meals. In Ghana, the children of mothers who were supplemented with SQ-LNS had improved birth size [4] and mean attained length at 18 mo [6] compared with the children of mothers who were provided the standard of care iron and folic acid (IFA) supplements during pregnancy [4,6,7]. The beneficial effect of SQ-LNS on child growth was greatest among children of primiparous mothers [4].

HDL particles are involved in many functions of relevance to maternal and child health, including protection from infectious pathogens [8,9]. HDL particles are most well studied for their role in reverse cholesterol transport, a key process to remove excess cholesterol from the body [10]. However, HDL particles are also well known to have a multitude of effects on immune cells [11]. For example, through their cholesterol efflux capacity (CEC), HDL particles enhance the macrophage-mediated response required to clear bacterial respiratory infection [9]. HDL functionality and concentrations are also negatively affected by inflammation and infection by a wide range of bacterial and viral pathogens [[12], [13], [14], [15], [16], [17]]. Plasma C-reactive protein (CRP) is a marker of inflammation and infection that has been associated with preterm delivery when concentrations are high [18]. CRP increases early during infection, whereas alpha-1-acid glycoprotein (AGP), an acute phase protein, increases later and remains elevated longer than CRP [19]. The combination of CRP and AGP can help identify individuals with a recent infection who do not yet show clinical symptoms (increased CRP) as well as those recovering from infections (increased AGP with or without increased CRP). Exploring the association between HDL function and these inflammatory markers during pregnancy may provide insight into how HDL function relates to different stages of inflammation during pregnancy and whether, in turn, these relationships are related to child birth outcomes.

However, HDL particles are heterogeneous, and their size, lipid, and protein components are known to influence their functional capacity [20,21]. HDL particles perform multiple pleiotropic functions, including anti-inflammatory and antioxidant functions, both of which are well established and are protective against oxidative stress, help maintain placental vascular function, and may influence pregnancy outcomes [22,23]. In Ghana, high HDL cholesterol (HDL-C) at 36 weeks of gestation was positively associated with a longer duration of gestation [24]. A recent study found maternal obesity was associated with impaired serum antioxidative capacity and lecithin-cholesterol acyltransferase (LCAT) activity but increased HDL CEC, further highlighting the potential role of HDL function in pregnancy [25]. This evidence suggests that maternal HDL function during pregnancy may be linked to early childhood growth and development.

HDL particles are highly dynamic and can be rapidly altered by diet. For example, our group has shown that just 4 d of dietary intervention alters the HDL lipidome [26]. Importantly, dietary interventions can influence the function of HDL particles without changing total HDL-C. For example, we and others have found that consumption of eggs improves HDL CEC without altering total HDL-C [27], highlighting the importance of assessing parameters beyond the simple measurement of HDL-C to understand the potential impact of diet on HDL. Although HDL particles have been extensively studied in the context of heart disease, little is known about HDL biology during pregnancy.

We have previously shown that long-term SQ-LNS supplementation from prenatal enrollment to 18 mo of age improved child HDL CEC [28]. The primary aim of this cross-sectional, secondary outcome analysis was to determine whether mothers supplemented with SQ-LNS have different measures of HDL functional capacity and metabolism compared to mothers supplemented with IFA among a subset of females at 36 weeks of gestation using archived samples from the previously conducted clinical study of the same mother-child dyad cohort. Seasonal variations in food availability can impact maternal nutritional status [29]. Infection rates also vary seasonally [30,31], and as mentioned previously, infection and inflammation are known to influence HDL concentration and function [[12], [13], [14], [15], [16], [17]]. Since HDL particles have a half-life of approximately 4 d in plasma [32], the function of HDL is highly dynamic and may be influenced by factors mediated by seasonal variation at the time of blood draw. Thus, we further explored whether these HDL functional parameters varied by season. Additional exploratory aims were associations between HDL CEC and enzyme activities with birth outcomes, including infant birth size and gestational duration, and maternal plasma inflammatory markers CRP and AGP. We hypothesized that mothers supplemented with SQ-LNS would have different HDL CEC as well as different activities of enzymes involved in HDL metabolism compared with mothers supplemented with IFA. We further hypothesized that HDL functional parameters would be associated with season at the time of blood draw, inflammation markers CRP and AGP, as well as pregnancy outcomes.

## Methods

### Participants

In this cross-sectional, secondary outcome analysis, we used archived samples from a previously conducted study in females (*N* = 1320, mean gestation age = 16.3 wk) who were enrolled in the iLiNS trial in Ghana to assess the effects of SQ-LNS on maternal and child growth outcomes [4,6]. The full study design, participant characteristics, and all relevant clinical trial information, including consent and Institutional Review Board documentation from the main trial, have been described in detail elsewhere [4]. Briefly, females were enrolled year-round during the wet (May – October) and the dry (November – April) seasons and were randomly assigned to receive either SQ-LNS, IFA, or multiple micronutrient supplements. Details of the 3 supplements have been described previously [4]. Project staff conducted biweekly visits to ensure fresh delivery of supplements and to monitor supplement consumption and maternal and child morbidity. Blood was drawn at 36 weeks of gestation, and plasma samples were separated at 1,252 × *g* for 15 min at room temperature and then stored at -33^o^C in Ghana before being airmailed on dry ice to Davis, CA, United States. Samples were stored at -80^o^C upon arrival.

**FIGURE 1:**
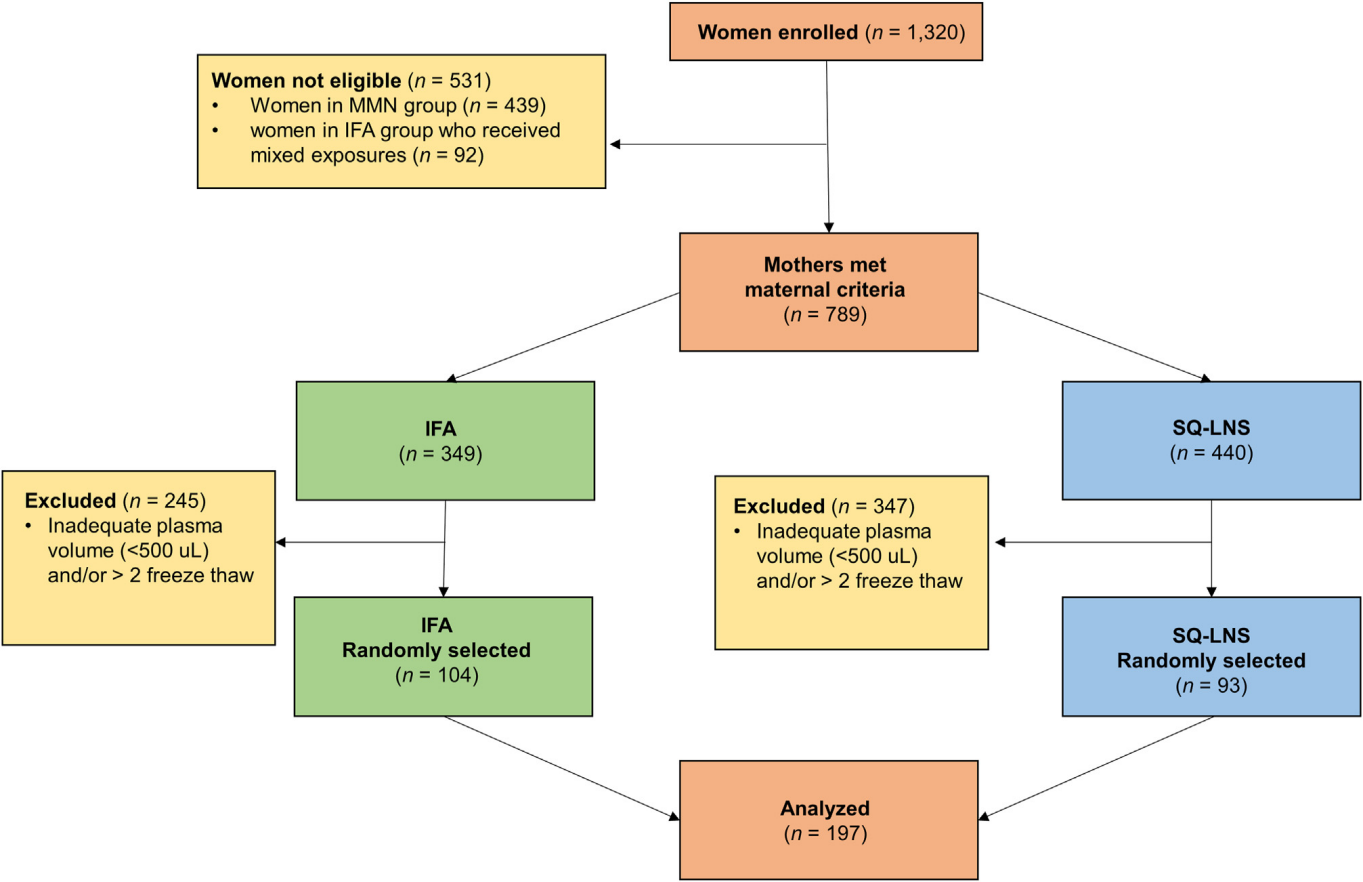
Study flow diagram. In this secondary outcome analysis, archived samples from a previously conducted study were utilized. A subset of participants were randomly selected from the original cohort as a representative sample set using the following criteria: the participant was randomly assigned to either SQ-LNS or IFA, enrolled between October 2010 and December 2011, adequate plasma volume (>500 μL), remained in the biorepository, and the sample was subjected to no more than 2 freeze-thaw cycles. IFA, iron and folic acid; SQ-LNS, small-quantity lipid-based nutrient supplements.

A study flow diagram is presented in Figure 1. In this subset of pregnant mothers, plasma samples from 197 females at 36 weeks of gestation were selected based on 1) being randomly assigned to either SQ-LNS or IFA (the multiple micronutrient group was excluded since difference in birth size was found to be statistically significant between the SQ-LNS and IFA groups, but not the multiple micronutrient group [4]), 2) enrollment between October 2010 and December 2011 to avoid the inclusion of females enrolled earlier who received a mixed exposure of supplements as previously described elsewhere [4], and 3) having adequate plasma volume from samples that were subjected to no more than 2 freeze-thaw cycles. Archived samples were collected and anonymized in September 2021 and then analyzed between September 2021 and March 2022. Characteristics of females, the assigned intervention group, and their infant birth anthropometrics were revealed after data collection.

The study protocol was approved by the ethics committees of the University of California, Davis; the Ghana Health Service; and the University of Ghana Noguchi Memorial Institute for Medical Research, and the main trial was registered at clinicaltrials.gov as NCT00970866. Written informed consent was obtained from all mothers prior to their participation, as detailed in the original publication detailing the main findings from the originally conducted study [4,6].

### Apolipoprotein B depletion

Plasma apolipoprotein B (ApoB) was precipitated by 20% polyethylene glycol (molecular weight 6000, Sigma-Aldrich catalog # 25322-68-3) in water as previously described by Davidson et al. [33]. Polyethylene glycol mixture (40 μL) was added to 100 μL plasma for 20 min at room temperature, followed by centrifugation at 10,000 × g for 30 min. The HDL-containing supernatant was aliquoted and stored at -80^o^C until used.

### HDL cholesterol efflux capacity

HDL CEC in ApoB-depleted plasma was measured using a commercially available kit (Abcam, Waltham, catalog # ab19685) as previously described [28,34] with modifications. J774A.1 (ATCC, catalog TIB-67) were seeded at 50,000 cells in 96-well plates for 18 h in Roswell Park Memorial Institute media containing 10% fetal bovine serum and 100 μg/mL penicillin and streptomycin. Cells were washed and labeled with fluorescent cholesterol along with acyl-CoA cholesterol acyltransferase inhibitor and cyclic adenosine monophosphate for 4 h. Cells were washed with FluroBrite Dulbecco's Modified Eagle Medium (Thermo Fisher Scientificcatalog # A1896701) followed by incubation with ApoB-depleted plasma (2%) in FluroBrite Dulbecco's Modified Eagle Medium for another 4 h. Cellular supernatant was removed, and the remaining cells were lysed with M-PER cell lysis buffer (Thermo Scientific, catalog # 78501) for 30 min. The fluorescence in the supernatant and lysed fraction was measured at 485/523 nm (emission/excitation) on a Synergy H1 plate reader (Biotek). The percentage of cholesterol efflux (i.e., CEC) was calculated as follows:

$$\%\;\text{cholesterol}\;\text{efflux}=\frac{\text{fluorescence}\;\text{intensity}\;\text{of}\;\text{the}\;\text{media}}{\text{fluorescence}\;\text{intensity}\;\text{of}\;\text{the}\;\text{cell}\;\text{lysate}+\text{media}}\times 100$$

### LCAT, PLTP, CETP activity assay

Commercially available kits were used to measure plasma LCAT, PLTP, and CETP activities (all from Roar Biomedical Inc.) in duplicate, following the manufacturer’s instructions with modifications.

LCAT activity (catalog # MAK107) measurements were collected on a Synergy H1 plate reader (BioTek) read at 340 nm excitation and 2 emission wavelengths at 390 nm and 470 nm, which represent the hydrolyzed and intact substrate, respectively. The increase in LCAT activity is indicated by increased λ_em_390 / λ_em_470 nm ratios.

CETP activity (catalog # MAK106) was measured in 20 μL of plasma (1:10 dilution in 10 mM Tris, 1 mM EDTA, 150 mM NaCl, pH 7.4) combined with synthetic cholesterol ester donor particles and lipoprotein acceptor particles provided by the kit. Plates were incubated for 3 h at 37^o^C and then measured at 465 nm excitation and 535 emission wavelengths on a Synergy H1 plate reader (Biotek). The increase in fluorescence intensity measures CETP's ability to catalyze the transfer of the fluorescent cholesterol ester from the donor to the acceptor particle.

PLTP activity (catalog # MAK 108) was measured in 50 μL of diluted plasma (1:10 dilution in 10 mM Tris, 1 mM EDTA, 150 mM NaCl, pH 7.4) by combining both donor and acceptor particles. Plates were incubated for 30 min at 37^o^C. The transferred fluorescence intensity was measured at 465 nm excitation and 535 emission wavelengths on a Synergy H1 plate reader (Biotek).

### Statistical analysis

The statistical analysis plan was posted before analysis (https://ilins.ucdavis.edu/). Sample size calculation is based on an effect size, calculated using Cohen's D, which represents a standardized measure of the difference between 2 means, of 0.4 from previous CEC data among children at 18 mo of age for mothers and their children enrolled in the iLiNS-DYAD trial in Ghana [28]. With a sample size of 100 per group, we can detect an effect size of 0.40 SDs in the mean difference between groups, assuming a 2-sided alpha of 0.05 and 80% power. Due to constraints in the availability of suitable samples (i.e., sample volume had to be >500 μL, and the sample was not subjected to >2 freeze-thaw cycles), 93 and 104 mothers from the SQ-LNS and IFA groups, respectively, were obtained. All data analyses were performed in R version 4.1.1 (R Project for Statistical Computing, Vienna, Austria). Data from females were analyzed based on an intention-to-treat basis where females were included regardless of adherence to the intervention. Variables were inspected for normality using the Shapiro-Wilks test. Non-normally distributed variables were naturally log-transformed first. Normalized ranks were created if the natural log-transformed data did not normalize the distribution.

Unadjusted and adjusted linear models were used to assess the effect of the intervention on HDL CEC and plasma LCAT, PLTP, and CETP activities. For the unadjusted model, a 2-sample t-test was used to compare intervention groups on normally distributed data. A Wilcoxon rank-sum test was performed on data that did not follow a normal distribution. We also performed unadjusted and adjusted linear models to compare the associations of HDL CEC or enzyme activities with birth outcomes, including infant weight, infant head circumference, infant length, and duration of gestation. Because plasma LCAT, PLTP, and CETP activities are not normally distributed, we report the differences in means in the unadjusted and adjusted models using the untransformed data but generated *P* values for group comparison using log-transformed data. The adjusted model included the following participant baseline characteristics if they were correlated with the outcome variable (Pearson *P* < 0.1): body mass index (BMI), age, years of formal education, height, gestational age at enrollment, season at enrollment (dry or wet), household food insecurity access score, asset index, parity, primiparous status, malaria status, maternal AGP, and maternal CRP, hemoglobin levels. We included both AGP and CRP as markers of inflammation to adjust linear models, given the known influence of inflammation on HDL function and metabolism [35,36]. The associations between intervention group and each HDL measure were considered statistically significant at *P* < 0.05. A chi-squared test or Fisher exact test was used to compare categorical outcome variables. Complete case analyses were performed to describe the characteristics of the study participants.

An exploratory analysis was performed to examine the associations between HDL CEC and plasma LCAT, PLTP, and CETP activities. For the association analysis, a Spearman’s test was used, adjusted for false discovery rate, and the R values were reported. We also explored whether these metrics were associated with inflammatory markers (AGP and CRP) and hemoglobin levels at 36 weeks of gestation. Lastly, we examined whether HDL CEC and plasma LCAT, PLTP, and CETP activities were associated with season at 36 weeks of gestation.

## Results

### Participant characteristics

Baseline characteristics of the full sample of females (*N* = 1320) are described previously [37]. Of the 1320 mothers enrolled in the full trial, 93 and 104 mothers from the SQ-LNS and IFA groups, respectively, were analyzed based on sample availability. This subset included those with adequate plasma volume (>500 μL) and no more than 2 freeze-thaw cycles to ensure sufficient material for the planned assays. The characteristics of the mothers (*N* = 197) at baseline and 36 weeks of gestation are presented in Table 1. There were no statistically significant differences in baseline or 36 weeks of gestation values between the SQ-LNS and IFA groups.

**TABLE 1:** Median (25th percentile, 75th percentile) characteristics of the study cohort of pregnant females in Ghana

Characteristics	IFA (*N* = 104)	SQ-LNS (*N* = 93)	*P*
≤20 weeks of gestation			
BMI, kg/m^2^	24.6 (22.0, 28.3) (*n =* 101)	24.8 (21.4, 27.9) (*n =* 92)	0.637
Maternal age, y	26.5 (24.0, 30.0) (*n =* 104)	26.0 (22.0, 30.0) (*n =* 93)	0.168
Education, completed years	9.0 (6.0, 9.0) (*n =* 104)	9.0 (6.0, 9.0) (*n =* 93)	0.938
Mother’s height, cm	158.7 (154.8, 162.2) (*n =* 101)	158.3 (155.7, 162.2) (*n =* 92)	0.729
Gestational age at enrollment, wk	16.9 (14.1, 18.7) (*n =* 104)	15.9 (13.9, 18.7) (*n =* 93)	0.389
Season at blood draw, dry^1^	49.0% (*n =* 104)	50.1% (*n =* 93)	0.834
Primiparous^1^ (%)	27.9% (*n =* 104)	34.4% (*n =* 93)	0.323
Parity (total births)	1.0 (0.0, 2.0) (*n =* 104)	1.0 (0.0, 2.0) (*n =* 93)	0.113
Household food insecurity access score	0.0 (0.0. 3.2) (*n =* 104)	0.0 (0.0. 2.0) (*n =* 92)	0.513
Asset index^2^	0.1 (-0.1, 0.9) (*n =* 104)	0.0 (-0.1, 0.6) (*n =* 93)	0.262
Overweight or obese 1 (% BMI ≥ 25 kg/m^2^)	46.5% (*n =* 101)	47.8% (*n =* 92)	0.858
Females with anemia 1 (Hb < 100 g/L)	8.7% (*n =* 104)	10.8% (*n =* 93)	0.618
Positive for malaria^13^	6.7% (*n =* 104)	6.5% (*n =* 93)	0.937
AGP (g/L)	0.6 (0.5, 0.8) (*n =* 99)	0.6 (0.5, 0.8) (*n =* 89)	0.857
CRP (mg/L)	4.0 (1.8, 8.7) (*n =* 99)	4.6 (1.6, 7.9) (*n =* 89)	0.564
36 weeks of gestation			
BMI, kg/m^2^	27.7 (25.1, 31.2) (*n =* 95)	27.5 (25.2, 30.9) (*n =* 89)	0.997
Season at blood draw, dry^1^	48.1% (*n =* 104)	53.8% (*n =* 93)	0.425
Females with anemia^1^ (Hb < 100 g/L)	2.9% (*n =* 104)	6.5% (*n =* 93)	0.231
Positive for malaria^13^	6.7% (*n =* 104)	6.5% (*n =* 93)	0.937
AGP (g/L)	0.4 (0.3, 0.5) (*n =* 104)	0.4 (0.3, 0.5) (*n =* 93)	0.535
CRP (mg/L)	2.1 (1.0, 4.5) (*n =* 104)	2.5 (1.1, 5.3) (*n =* 93)	0.456

AGP, alpha-1-acid glycoprotein; BMI, body mass index; CRP, C-reactive protein; IFA, iron and folic acid; SQ-LNS, small-quantity lipid-based nutrient supplement.

^3^Rapid diagnostic test by Clearview Malarial Combo, Vision Biotech.

1 Chi-square test for indicated categorical variables.

2 Proxy indicators for household socioeconomic status.

### Cross-sectional analysis of HDL functional measures in SQ-LNS supplemented mothers and associations with birth outcomes

The primary HDL outcome variables are shown in Table 2. HDL CEC and plasma LCAT, PLTP, and CETP activities were not statistically significantly different by intervention group in the unadjusted or the adjusted model (*P* > 0.05 for all).

**TABLE 2:** Primary HDL CEC and enzyme activities by supplement group at 36 weeks of gestation

Outcome	Results by study group		Unadjusted Model		Adjusted Model^3^	
	IFA (*N* = 104)	SQ-LNS (*N* = 93)	Difference in means (95% CI)	*P*	Difference in means (95% CI)	*P*
Cholesterol efflux capacity^1^ (%)	38.1 ± 4.8	37.7 ± 4.5	-0.4 (-1.7, 0.9)	0.570	-0.2 (-1.5, 1.2)	0.821
LCAT activity^2^ (390/470 nm ratio)	1.4 [1.3, 1.5]	1.4 [1.3, 1.5]	0.03 (-0.01, 0.07)	0.182	0.02 (-0.02, 0.06)	0.320
PLTP activity^2^ (pmol/μL/h)	97.5 [66.3, 134.0]	101.5 [68.0, 147.6]	3.0 (-12.9, 18.9)	0.681	2.7 (-13.3, 18.7)	0.705
CETP activity^2^ (pmol/μL/h)	26.6 [18.0, 36.6]	26.7 [15.8, 36.2]	0.7 (-3.3, 4.6)	0.995	-0.08 (-4.0, 3.8)	0.773

CETP, cholesteryl ester transfer protein; IFA, iron and folic acid; LCAT, lecithin-cholesterol acyltransferase; PLTP, phospholipid transfer protein; SQ-LNS, small-quantity lipid-based nutrient supplement.

1 Values are represented as mean ± SDs, 2-tailed parametric test.

2 Values are represented as median [25th percentile, 75th percentile], 2-tailed nonparametric test. Data are presented as raw values, but the statistical analysis of both the unadjusted and adjusted models was performed using log-transformed data to better approximate normal distribution.

3 The adjusted model included the mother’s baseline characteristics if the outcome variables were Pearson correlated (*P* < 0.1), which includes body mass index (BMI), age, years of education, height, gestational age, season, parity, primiparous, household food insecurity access score, asset index, malaria status, alpha-1-acid glycoprotein (AGP) and C-reactive protein (CRP), and hemoglobin levels.

We examined whether there were any associations between any of the HDL functional measures and birth outcomes or duration of gestation. None of the HDL variables were statistically significantly correlated with infant birth weight, infant head circumference, infant length, or duration of gestation in the unadjusted or adjusted models (*P* > 0.05, Table 3).

**TABLE 3:** Correlation of birth outcomes with HDL CEC and enzyme activities at 36 weeks of gestation

Outcome	Unadjusted		Adjusted^1^	
	β (SE)	*P*	β (SE)	*P*
Infant birth weight (kg)				
Cholesterol efflux capacity (%)	1.0 (6.0)	0.958	1.0 (6.0)	0.920
LCAT activity^2^ (390/470 nm ratio)	-0.2 (0.2)	0.400	-0.1 (0.02)	0.689
PLTP activity^2^ (pmol/μL/h)	-0.2 (0.5)	0.454	-0.3 (0.4)	0.353
CETP activity^2^ (pmol/μL/h)	-2.0 (2.0)	0.493	-0.1 (2.0)	0.842
Infant head circumference at birth (cm)				
Cholesterol efflux capacity (%)	0.03 (0.02)	0.124	0.03 (0.02)	0.125
LCAT activity^2^ (390/470 nm ratio)	-0.3 (0.6)	0.557	0.4 (0.6)	0.536
PLTP activity (pmol/μL/h)	0.2 (2.0)	0.833	-0.9 (1.0)	0.477
CETP activity (pmol/μL/h)	-8.0 (6.0)	0.166	3.0 (6.0)	0.950
Infant length at birth (cm)				
Cholesterol efflux capacity (%)	-0.02 (0.02)	0.367	-0.03 (0.03)	0.223
LCAT activity^2^ (390/470 nm ratio)	0.5 (0.5)	0.471	0.5 (0.8)	0.505
PLTP activity^2^ (pmol/μL/h)	-2.0 (2.0)	0.404	-2.0 (2.0)	0.347
CETP activity^2^ (pmol/μL/h)	-3.0 (8.0)	0.596	8.0 (9.0)	0.216
Duration of gestation (wk)				
Cholesterol efflux capacity (%)	0.02 (0.02)	0.408	0.02 (0.02)	0.397
LCAT activity^2^ (390/470 nm ratio)	0.3 (0.6)	0.542	0.7 (0.6)	0.284
PLTP activity^2^ (pmol/μL/h)	0.9 (2.0)	0.580	0.6 (1.0)	0.588
CETP activity^2^ (pmol/μL/h)	-10.0 (6.0)	0.223	-7.0 (7.0)	0.533

1 The adjusted model included the mother’s baseline characteristics if the outcome variables were Pearson correlated (*P* < 0.1), which includes body mass index (BMI), age, years of education, height, gestational age, season, parity, primiparous, household food insecurity access score, asset index, malaria status, alpha-1-acid glycoprotein (AGP) and C-reactive protein (CRP), and hemoglobin levels. LCAT, lecithin-cholesterol acyltransferase; PLTP, phospholipid transfer protein; CETP, cholesteryl ester transfer protein.

2 Data are presented as raw values, but the statistical analysis of both the unadjusted and adjusted models were performed using log-transformed data to better approximate normal distribution.

### Correlations between HDL CEC and enzyme activities

**FIGURE 2:**
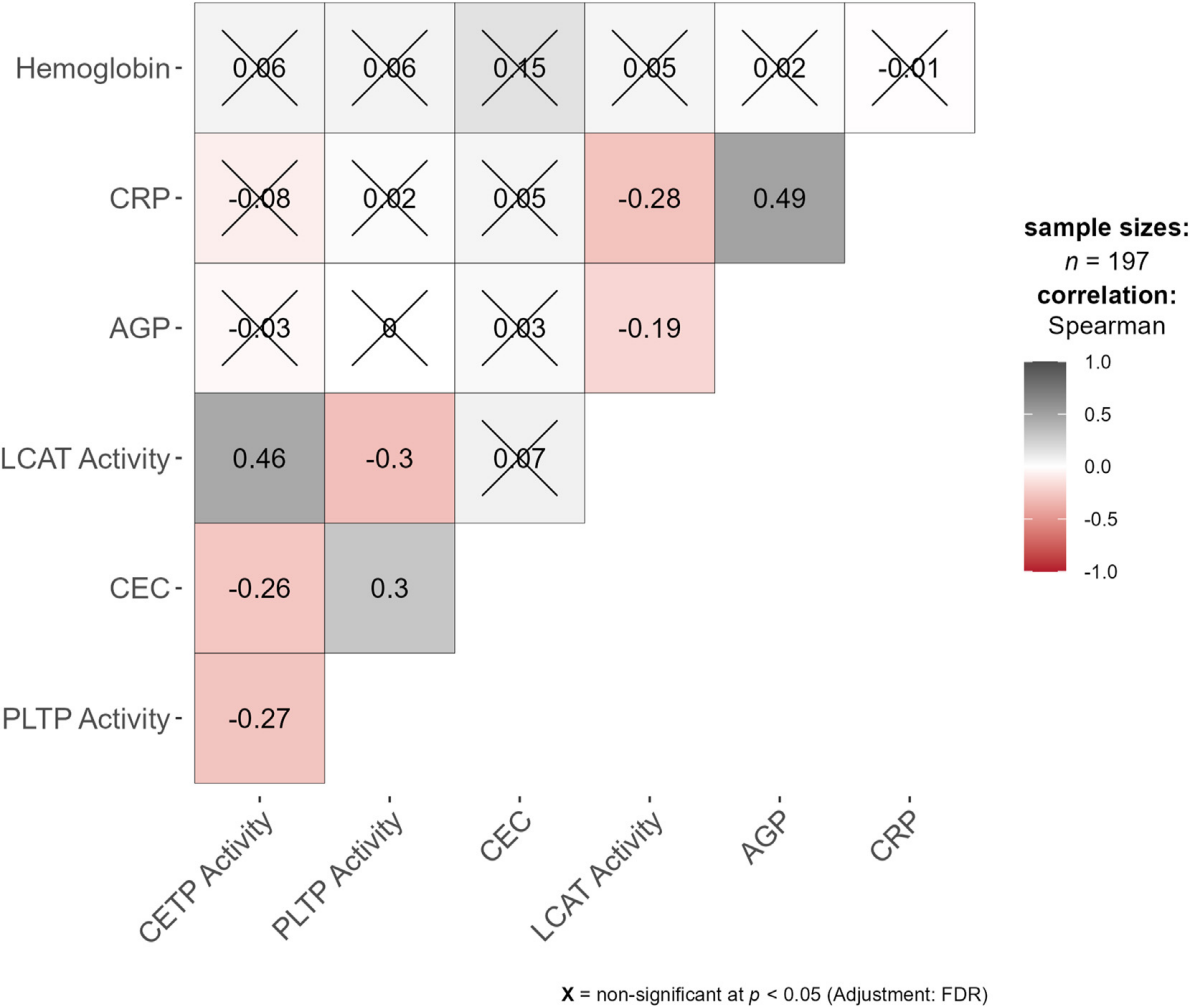
Spearman correlation analysis between HDL CEC, plasma enzyme activities, inflammation markers, and hemoglobin levels. AGP, alpha-1-acid glycoprotein; CEC, cholesterol efflux capacity; CETP, cholesteryl ester transfer protein; CRP, C-reactive protein; LCAT, lecithin-cholesterol acyltransferase; PLTP, phospholipid transfer protein.

In this cross-sectional analysis, our primary analysis did not support our hypothesis that mothers supplemented with SQ-LNS would have different HDL CEC and LCAT, PLTP, and CETP activities compared with mothers supplemented with IFA control at 36 weeks of gestation. The secondary objective was to explore potential associations among HDL CEC and plasma LCAT, PLTP, and CETP activities, independent of the intervention, to further elucidate the mechanisms influencing these variables. We examined whether HDL CEC and plasma LCAT, PLTP, and CETP activities were associated regardless of intervention (Figure 2). HDL CEC was statistically significantly negatively correlated with CETP activity (R = -0.26, 95% confidence interval [CI] (-0.39, -0.12), *P* < 0.001) and positively correlated with PLTP activity (R = 0.30, 95% CI (0.17, 0.43), *P* < 0.001). CETP activity was statistically significantly positively correlated with LCAT activity (R = 0.46, 95% CI (0.33, 0.56), *P* < 0.001). Both CETP and LCAT activities were statistically significantly negatively correlated with PLTP activity (R = -0.27, 95% CI (-0.40, -0.13), *P* < 0.001; R = -0.30, 95% CI (-0.43, -0.16), *P* <0.001, respectively).

### Associations between HDL functional measures and inflammatory markers and hemoglobin levels

We explored whether HDL CEC and plasma LCAT, PLTP, and CETP activities were correlated with inflammatory markers AGP and CRP, as well as hemoglobin concentrations at 36 weeks of gestation (Figure 2). AGP and CRP were statistically significantly positively correlated (R = 0.49, 95% CI (0.37, 0.59), *P* < 0.001). LCAT activity was statistically significantly negatively correlated with both AGP (R = -0.19, 95% CI (-0.33, -0.05), *P* = 0.007) and CRP (R = -0.28, 95% CI (-0.41, -0.14), *P* < 0.001), whereas HDL CEC, PLTP activity, and CETP activity were not statistically significantly correlated with either AGP or CRP (*P* > 0.05). HDL CEC was positively correlated with hemoglobin (R = 0.15, 95% CI (0.01, 0.29), *P* = 0.036), but this association was not statistically significant after correction for multiple testing. There were no statistically significant correlations observed between plasma LCAT, PLTP, or CETP activities and hemoglobin.

### HDL enzyme activities differ by season

**FIGURE 3:**
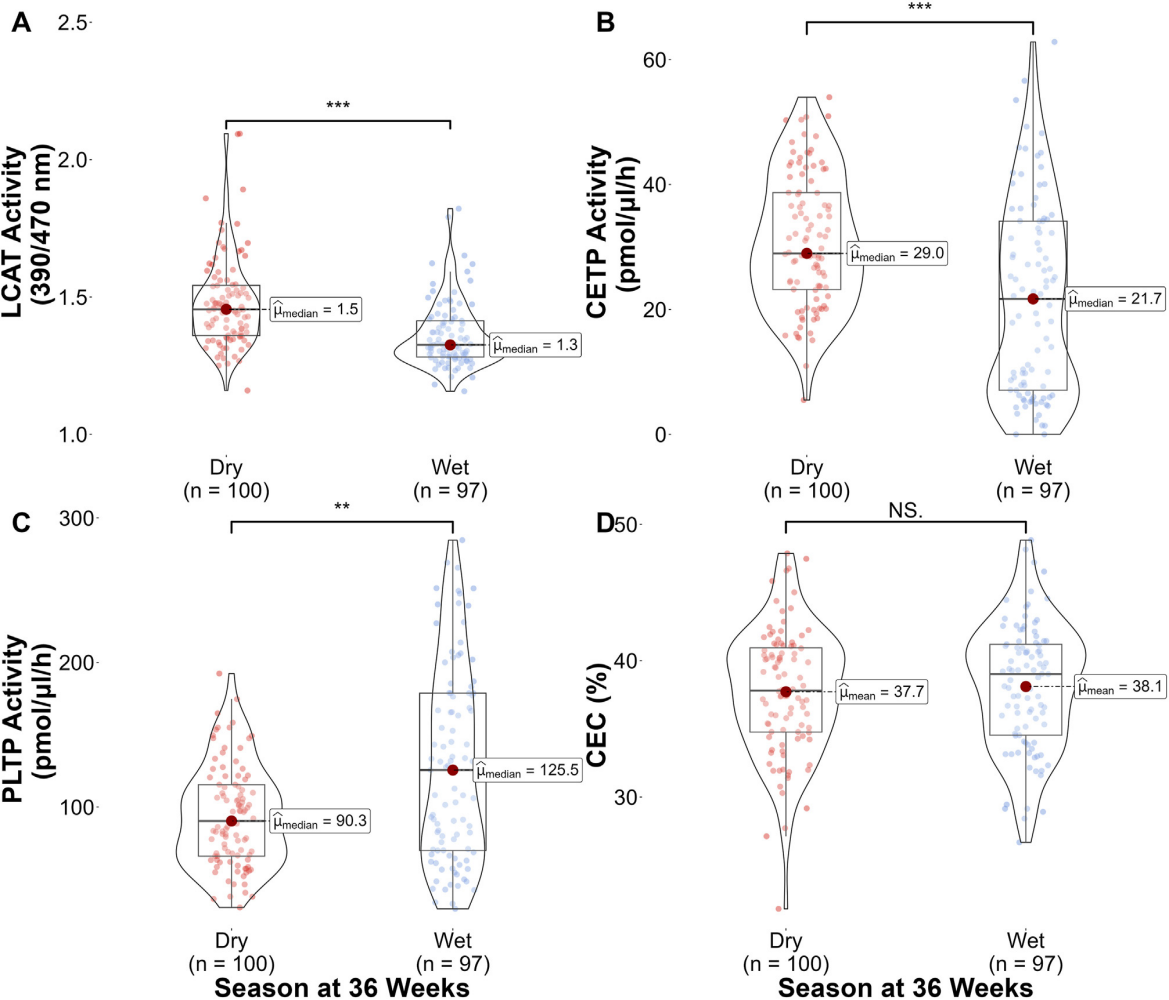
Box and violin combination plots of plasma enzyme activities and HDL cholesterol efflux capacity (CEC) by season (Dry: November - April and Wet: May - October) at 36 weeks of gestation. Plasma (A) lecithin-cholesterol acyltransferase (LCAT) activity, (B) cholesteryl ester transfer protein (CETP) activity, (C) phospholipid transfer protein (PLTP) activity, and (D) HDL CEC. ∗∗*P* < 0.01, ∗∗∗*P* < 0.001, NS = not statistically significant.

We determined whether HDL CEC and plasma LCAT, PLTP, and CETP activities were associated with season at 36 weeks of gestation. Regardless of intervention group, median (25th percentile, 75th percentile) LCAT activity was statistically significantly higher in the dry season compared to the wet season (1.5 [1.4, 1.5] vs. 1.3 [1.3, 1.4] 390/470 nm, *P* < 0.001) and CETP activity was statistically significantly higher in the dry season compared to the wet season (29.0 (23.2, 38.7) vs. 21.7 (7.1, 34.1) pmol/μL/h, *P* < 0.001) (Figure 3AB). By contrast, PLTP activity was statistically significantly higher during the wet season compared to the dry season (125.5 (69.9, 178.7) vs. 90.3 (66.0, 115.5) pmol/μL/h, *P* = 0.001 Figure 3C). HDL CEC was not statistically significantly different by season (*P* = 0.560, Figure 3D). We further explored whether changes in enzyme activities were driven by inflammation. There were no statistically significant differences in positive rate of malaria, AGP, and CRP by season at 36 weeks of gestation (*P* > 0.05, Supplemental Table 1).

## Discussion

In this cross-sectional, secondary outcome analysis, we explored whether there were differences in HDL CEC and plasma enzymes involved in HDL metabolism, measured at 36 weeks of gestation, between mothers supplemented with SQ-LNS and those mothers supplemented with IFA enrolled in the iLiNS-DYAD trial in Ghana. We found that mothers supplemented with SQ-LNS from enrollment (≤ 20 wk) to 36 weeks of gestation did not have statistically significantly different HDL CEC or plasma LCAT, PLTP, and CETP activities compared to mothers supplemented with the IFA control at 36 weeks of gestation, and none of these were statistically significantly associated with pregnancy or birth outcomes.

Both CEC and LCAT activity are measures of the ability of HDL to perform reverse cholesterol transport, involved in the removal of excess cholesterol from the body. LCAT is involved in the process of HDL maturation, in which cholesterol molecules are esterified and sequestered in the core of the particle, thus enabling higher total cholesterol carrying capacity [38]. Importantly, LCAT is also involved in anti-infectious functions, as it is required for the ability of HDL particles to bind lipopolysaccharide [39]. Likewise, higher PLTP activity was found to be associated with lower levels of LPS in plasma in cardiac surgery patients, which suggests that PLTP is also involved in lipopolysaccharide elimination [40]. By contrast, CETP is inhibited in an infectious state [36], which has been suggested to prevent the deleterious removal of HDL and to improve bacterial clearance [41]. Together, LCAT, PLTP, and CETP activities are associated with infection by decreasing PLTP activity and increasing both LCAT and CETP activities, which in turn can alter HDL metabolism [15,36)]. CETP can transfer cholesteryl esters formed by LCAT from HDL to triglyceride-rich lipoproteins, including very low-density lipoproteins and low-density lipoproteins, in exchange for triglycerides [42]. Both the activities of CETP and LCAT have been shown to increase during pregnancy, and both are associated with increased concentrations of maternal plasma lipids [43,44]. PLTP mediates the transfer of phospholipids from triglyceride-rich lipoproteins to HDL particles, and its activity is critical in part for the transfer of cholesterol from mother to fetus [45]. It is possible that the lack of observed differences in HDL CEC and enzyme activities between the SQ-LNS and IFA groups is due to the natural changes to lipoproteins during pregnancy, which may have obscured the possible effects of SQ-LNS on these functional parameters.

We reported previously that HDL CEC of children at 18 mo of age assigned to the SQ-LNS group was increased compared with children in the IFA group (28). ALA, an essential n-3 polyunsaturated fatty acid and one of the nutrients provided in the SQ-LNS, has been shown to improve HDL CEC in vitro [46]. In this cohort, at 36 weeks of gestation the mothers had higher ALA: DHA ratios in the SQ-LNS group compared with the IFA group, but the median DHA concentration in plasma, as well as overall plasma fatty acids and lipid concentrations, were not statistically significantly different [47]. The lack of difference in HDL CEC between the SQ-LNS and IFA groups at 36 weeks of gestation could be due to the lack of difference in fatty acid profiles in the 2 groups or to other unknown factors.

The lack of association between HDL functional measures and birth outcomes was somewhat surprising. Among a subset of 320 out of 1320 females in this trial, total HDL-C was positively associated with the duration of gestation [24]. Even so, HDL CEC is recognized as a better indicator of health risk than HDL-C alone across many cohorts [[48], [49], [50]]. Preeclampsia patients have elevated plasma CEC but lower ATP-Binding Cassette Subfamily A Member 1 (ABCA1)-mediated CEC, compared with plasma from normotensive pregnant females, which the authors postulate as a rescue mechanism in preeclampsia to mitigate lipid peroxidation [51]. In a cohort of Scandinavian females (*N* = 1031), low plasma CETP activity at 36 to 38 weeks of gestation was associated with giving birth to small-for-gestational-age infants but not associated with large-for-gestational-age infants, which suggests CETP activity may be related to fetal growth restriction [52]. More studies are needed to better understand the mechanisms by which HDL functional measures may influence pregnancy and birth outcomes.

We found that plasma CETP and LCAT activities were positively correlated but were both negatively correlated with PLTP activity, whereas CETP activity was additionally negatively correlated with HDL CEC. LCAT activity was found to be negatively correlated with concentrations of the inflammatory markers AGP and CRP at 36 weeks of gestation, which is in agreement with other observations of reduced LCAT activity during inflammation [15,36,53]. HDL CEC and plasma PLTP activity were positively correlated, and this association was also observed in participants with and without metabolic syndrome [54]. Others have linked higher PLTP activity with an improved ability of HDL to efflux cholesterol [55,56] through its ability to stabilize and interact with ABCA1 (56). Moreover, PLTP participates in the reverse cholesterol transport pathway across the fetal placental barrier [57]. By contrast, high PLTP activity has been linked to inflammation, particularly in sepsis patients [15,36]. These studies suggest that the association between the enzymes involved in lipoprotein metabolism differs across health statuses and further work is needed to clarify the relationship between HDL function and metabolism in the context of pregnancy. Longitudinal sampling throughout the course of pregnancy would be particularly informative to understand how HDL metabolism changes during normal pregnancy and in pregnancies in which infection episodes or other inflammatory factors are prevalent.

We observed that both plasma CETP and LCAT activities were higher among participants in the dry season at 36 weeks of gestation, whereas PLTP activity was higher during the wet season in this cohort of females in Ghana. Although we observed an association between LCAT and both AGP and CRP, we did not observe a statistically significant difference in AGP or CRP levels or differences in the proportion with a positive rapid test for malaria by season, which suggests that inflammation was not the primary factor driving seasonal differences in these enzymes. In the wet season, there is higher consumption of vitamin A-rich fruits, including mangoes, and vitamin A-rich dark leafy green vegetables among children in northern Ghana [58], which suggests that seasonality can affect the availability of certain foods, influence nutrient status, and possibly HDL metabolism. Together, these seasonal associations with plasma enzymes could be mediated by a combination of factors, including variation in inflammation, infection prevalence, or food availability.

This study had limitations. We used cross-sectional samples, which restricts our ability to detect changes in HDL function over time. Furthermore, there was a lack of evidence to support the observed association between season and plasma enzyme activities. Thus, we cannot conclusively explain the observed differences. Plasma HDL-C and ApoA-I were not measured in this study. However, these measures have been shown to be only modestly correlated with CEC across large cohorts [49,50]. Lastly, this subsample analyzed based on sample availability criteria for volume and freeze-thaw is not likely representative of the full sample enrolled in the trial. The constraints of adequate plasma volume and limited freeze-thaw cycles introduced potential selection bias into the analyzed sample, and thus, these findings may not be generalizable to the entire study population. Further research in larger robust sample sets is warranted to confirm the relationships identified here.

In summary, we did not observe differences in HDL CEC or plasma LCAT, PLTP, or CETP activities at 36 weeks of gestation between mothers supplemented with SQ-LNS compared with those in the IFA control group. Maternal HDL function was also not associated with childbirth outcomes. However, seasonal factors (e.g., changes in food availability, infection rates) and markers of inflammation were associated with HDL function, indicating that these factors had a stronger influence on HDL functionality than SQ-LNS supplementation during pregnancy in this cohort of females in Ghana.

## Author contributions

The authors’ responsibilities were as follows—SA, HO, AL, and KGD participated in designing and conducting the original study. BVH, AMZ, and KGD designed the secondary outcome analysis. BVH, JJZ, EZR, and JKA analyzed the biological samples. BVH, XT, and CDA analyzed the data statistically. BVH, AMZ, and KGD wrote the manuscript. All authors read and approved the final manuscript.

## Conflict of interest

The authors declare that the research was conducted in the absence of any commercial or financial relationships that could be construed as a potential conflict of interest.

## Funding

The project described was supported by Bill & Melinda Gates Foundation grant (OPP124589) to the University of California, Davis. The findings and conclusions contained within this work are solely the responsibility of the authors and do not necessarily represent the official views of the Bill & Melinda Gates Foundation.

## Data availability

The original contributions presented in the study are included in the supplementary material; further inquiries can be directed to the corresponding author.
